# Evaluation of rapid transepithelial electrical resistance (TEER) measurement as a metric of kidney toxicity in a high-throughput microfluidic culture system

**DOI:** 10.1038/s41598-022-16590-9

**Published:** 2022-08-01

**Authors:** Erin M. Shaughnessey, Samuel H. Kann, Hesham Azizgolshani, Lauren D. Black, Joseph L. Charest, Else M. Vedula

**Affiliations:** 1 The Charles Stark Draper Laboratory Inc, 555 Technology Square, Cambridge, MA 02139 USA; 2 Department of Biomedical Engineering, Tufts University, 4 Colby Street, Medford, MA 02155 USA; 3 Department of Mechanical Engineering, Boston University, 110 Cummington Mall, Boston, MA 02215 USA

**Keywords:** Tissue engineering, High-throughput screening

## Abstract

Rapid non-invasive kidney-specific readouts are essential to maximizing the potential of microfluidic tissue culture platforms for drug-induced nephrotoxicity screening. Transepithelial electrical resistance (TEER) is a well-established technique, but it has yet to be evaluated as a metric of toxicity in a kidney proximal tubule (PT) model that recapitulates the high permeability of the native tissue and is also suitable for high-throughput screening. We utilized the PREDICT96 high-throughput microfluidic platform, which has rapid TEER measurement capability and multi-flow control, to evaluate the utility of TEER sensing for detecting cisplatin-induced toxicity in a human primary PT model under both mono- and co-culture conditions as well as two levels of fluid shear stress (FSS). Changes in TEER of PT-microvascular co-cultures followed a dose-dependent trend similar to that demonstrated by lactate dehydrogenase (LDH) cytotoxicity assays and were well-correlated with tight junction coverage after cisplatin exposure. Additionally, cisplatin-induced changes in TEER were detectable prior to increases in cell death in co-cultures. PT mono-cultures had a less differentiated phenotype and were not conducive to toxicity monitoring with TEER. The results of this study demonstrate that TEER has potential as a rapid, early, and label-free indicator of toxicity in microfluidic PT-microvascular co-culture models.

## Introduction

Identification of organ-specific toxicity remains a challenge for drug development, contributing to high rates of attrition and rising costs of approved therapies^1^. Acute kidney injury is one of the most commonly reported drug-induced complications and typically manifests as damage to the proximal tubule (PT)^2^. In vitro kidney models consisting of a single cell type in static culture fail to predict drug-induced nephrotoxicity due to poor replication of the native microenvironment^3^. Emerging microfluidic models incorporate physiological stimuli, such as fluid shear stress (FSS) and heterogeneous cell populations, and show promise for reducing the burden of unidentified toxicities by better replicating native tissue structure and function compared to existing preclinical models^4,5^. To fully realize the benefits of microfluidic kidney models in the drug development pipeline and deepen understanding of nephrotoxicity mechanisms, novel platforms must facilitate efficient screening of culture conditions and provide robust methods for evaluating cellular responses^3^.

Microfluidic culture systems readily accommodate fluid flow and enable spatially controlled co-culture of multiple cell types, making this technology highly relevant for nephrotoxicity screening applications^6^. FSS has been shown to influence PT tissue structure^7,8^, metabolism^9,10^, and transport function^11,12^ in vitro relative to static conditions and is thus an important factor to consider in toxicity screening assays. Similarly, communication with microvascular endothelial cells has been found to impact the transcriptional regulation of PT transport-associated genes^13,14^, highlighting the importance of multiple cell types in models for kidney toxicity evaluation. High-throughput platforms enable simultaneous evaluation of various dosing schemes and proper controls as well as different culture conditions, such as multiple cell types and varying levels of FSS. Microfluidic tissue models with high throughput capability thus have the potential to provide a more complete view of how nephrotoxicity is influenced by the microenvironment^15^.

Metrics of PT function that can be probed repeatedly and non-invasively on the same tissue have the potential to provide new insights into the pathophysiology of PT toxicity. Heavy reliance on cytotoxicity endpoint assays limits the extent to which dynamic, drug-induced responses can be characterized in high-throughput models^3,6^. Several studies have demonstrated changes in PT barrier function in response to toxic compounds^16–18^. One approach to characterize changes in PT barrier properties in vitro is a permeability assay, which measures the flux of fluorescent tracer molecules across a tissue barrier providing information about paracellular fluid flow and tight junction pore size^19^. However, in addition to being labor-intensive, the results of these assays reflect the permeability of tracers with discrete molecular size, and they are typically limited to endpoint use because tissues must be exposed to exogenous reagents^19^.

To facilitate repeated interrogation of tissue replicates, non-invasive sensing technologies have been developed to enable rapid detection of changes in tissue structure and function^20^, and several modalities have been recently implemented in kidney in vitro models^21,22^. Transepithelial electrical resistance (TEER) is a well-established, non-invasive readout that reflects the ionic conductance of the paracellular pathway providing information about barrier integrity and permeability^19^. TEER sensing has previously been implemented in Transwell models and singular microfluidic chips to characterize PT barrier formation and to detect PT response to nephrotoxic compounds^23–25^. For example, previous researchers have used TEER sensing to evaluate PT response to cisplatin, a well characterized nephrotoxin, in static cultures of immortalized PT cells, such as RPTEC/TERT1^16,26^. While kidney cell lines have the advantage of low batch-to-batch variability, they exhibit significantly greater TEER than primary PT cells^27,28^, so it is unclear whether their barrier function profiles recapitulate toxicity-induced changes in vivo. Models that reproduce the leakiness of PT epithelia using primary cells may be more suitable candidates for a TEER-based assay of toxicity ^3^. However, TEER sensing has yet to be investigated in a toxicity model with in-vivo-like PT barrier function that could be scaled to meet preclinical screening needs.

In this paper, we explore the ability to detect drug-induced kidney toxicity in primary PT cells using TEER sensing in a high-throughput model, while screening the effects of two flow conditions and microvascular co-culture. PREDICT96 is a high-throughput microfluidic system with rapid TEER measurement capability that enables parallel culture of 96 independent tissue replicates and control of multiple fluid flows within an industry-standard well-plate footprint^29^. We utilized PREDICT96 to develop a model of the PT epithelia using human primary cells which demonstrate features of native morphology when co-cultured with human primary microvascular endothelial cells under continuous FSS, hypothesizing that the co-culture environment would yield a model with greater potential to predict in vivo toxicity. Here, we evaluate co-culture and mono-culture models where PT cells are exposed to a high FSS, mimicking physiological levels^8^, and a low FSS using a single microfluidic culture plate. We demonstrate that while PT mono-cultures have similar baseline TEER to co-cultures, they lack markers of differentiation and show significantly higher levels of baseline cell death. We observe that cisplatin induces dose-dependent toxicity and tight junction loss in both models but only PT cells in the co-culture model exhibits dose-dependent changes in TEER that align with cytotoxicity and tight junction readouts. Additionally, we find that TEER readouts signal the onset of cisplatin toxicity at earlier time points than increases in cell death. The results of our study demonstrate that TEER is an indicator of cisplatin toxicity in the PREDICT96 co-culture kidney model with the potential to improve nephrotoxicity detection efficiency.

## Results

### High-throughput microfluidic platform supports human primary cell model of the kidney proximal tubule (PT)

**Figure 1:**
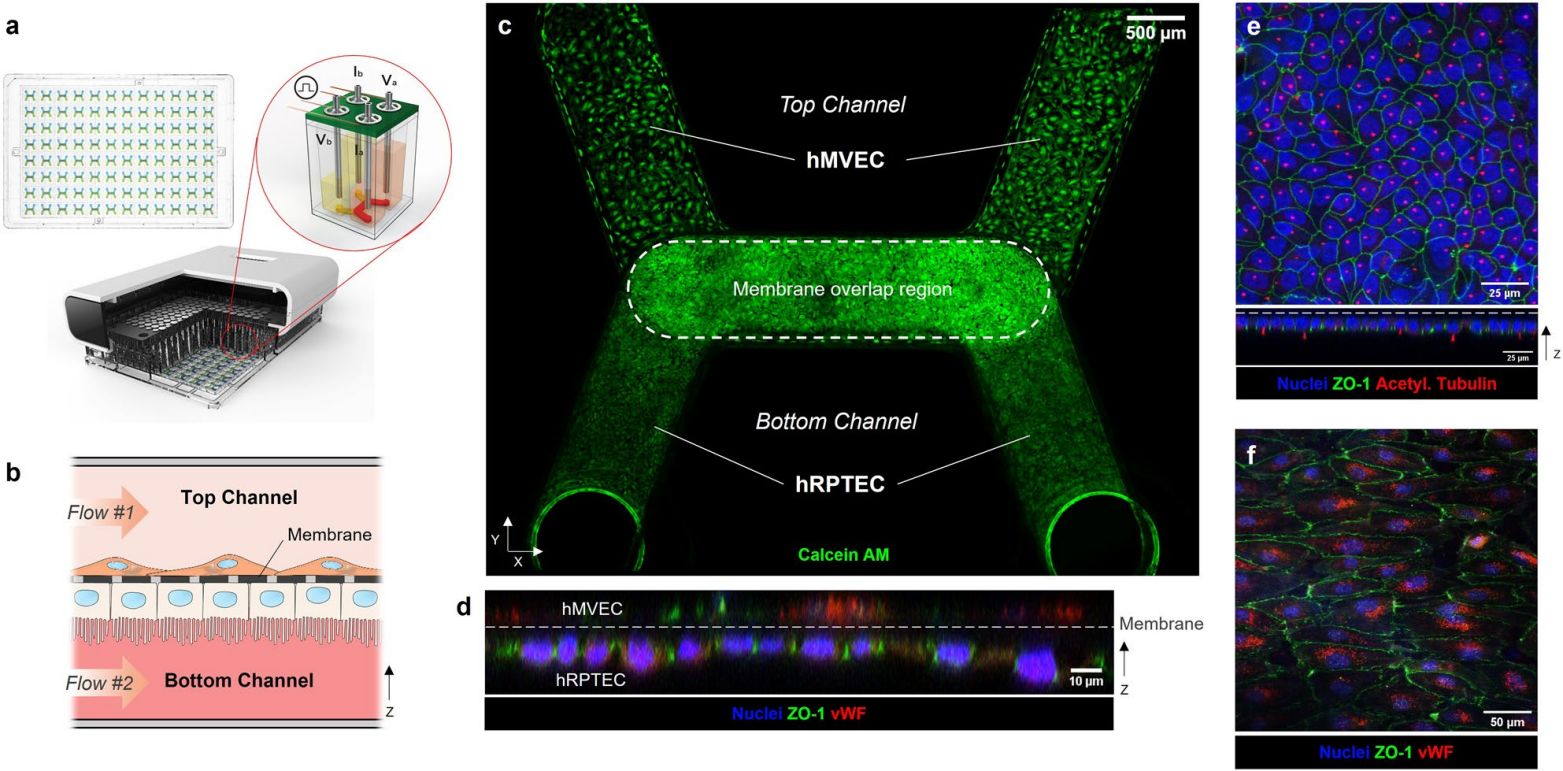
The PREDICT96 platform with integrated TEER sensing supports proximal tubule-microvascular co-culture with in-vivo-like features. (**a**) The PREDICT96 culture plate has 96 microfluidic devices (top left). Cross-sectional rendering of PREDICT96 Integrated TEER system (bottom) highlights the four-point TEER measurement unit (illustration, top right) in which the stainless steel pump tubes double as electrodes. (**b**) A cross-section schematic of the co-culture kidney model in the bilayer microfluidic device with hRPTEC on bottom of the membrane and hMVEC on top of the membrane. Fluid flow is controlled separately in top and bottom channels. (**c**) A confocal tile scan of a PREDICT96 device shows confluent cell layers under high FSS (0.70 dyn/cm^2^) on day 7 (Calcein AM, green). (**d**) An orthogonal view of a confocal z-stack shows hRPTEC and hMVEC on either side of the device membrane (dashed line) with hRPTEC expressing apical ZO-1 (green) and hMVEC expressing endothelial marker von Willebrand factor (vWF, red). (**e**, top) A maximum intensity projection of a z-stack of hRPTEC on the bottom side of the membrane shows continuous tight junctions (ZO-1, green) and abundant primary cilia (acetylated tubulin, red). (**e**, bottom) An orthogonal view demonstrates hRPTEC apical expression of ZO-1 and primary cilia. (**f**) A confocal z-slice of hMVEC on the top side of the membrane shows characteristic punctate expression of vWF (red) and tight junctions (green).

The PREDICT96 TEER system enables rapid interrogation of tissue barrier function across 96 replicate microfluidic devices (Fig. 1a). To evaluate TEER as a metric of kidney toxicity, we developed a model of the proximal tubule in the PREDICT96 system. Human primary renal proximal tubule epithelial cells (hRPTEC) and human primary microvascular endothelial cells (hMVEC) were cultured on the bottom and top side of the porous membrane, respectively, creating a co-culture model of the PT with independent fluid flow in each channel (Fig. 1b). Both cell types formed viable tissue layers in the microfluidic device visible by live Calcein AM staining (Fig. 1c). Confocal z-stacks confirmed that hRPTEC and hMVEC occupied either side of the permeable membrane in the overlap region under co-culture conditions, illustrating the potential for cellular crosstalk (Fig. 1d). Low level off-target staining for von Willebrand factor (vWF) in hRPTEC is visible and likely due to sequence similarities with the polyclonal antibody. Within 5 to 7 days of culture in PREDICT96 under high FSS (0.70 dyn/cm^2^), hRPTEC developed markers of polarization including tight junctions that localize to the edge of the apical membrane as well as primary cilia that can be seen protruding from the cell surface (Fig. 1e). Co-cultured hMVEC also exposed to high FSS expressed endothelial marker vWF and formed tight junctions (Fig. 1f).

### PREDICT96 facilitates simultaneous characterization of mono- and co-culture PT tissue under different flow conditions

**Figure 2:**
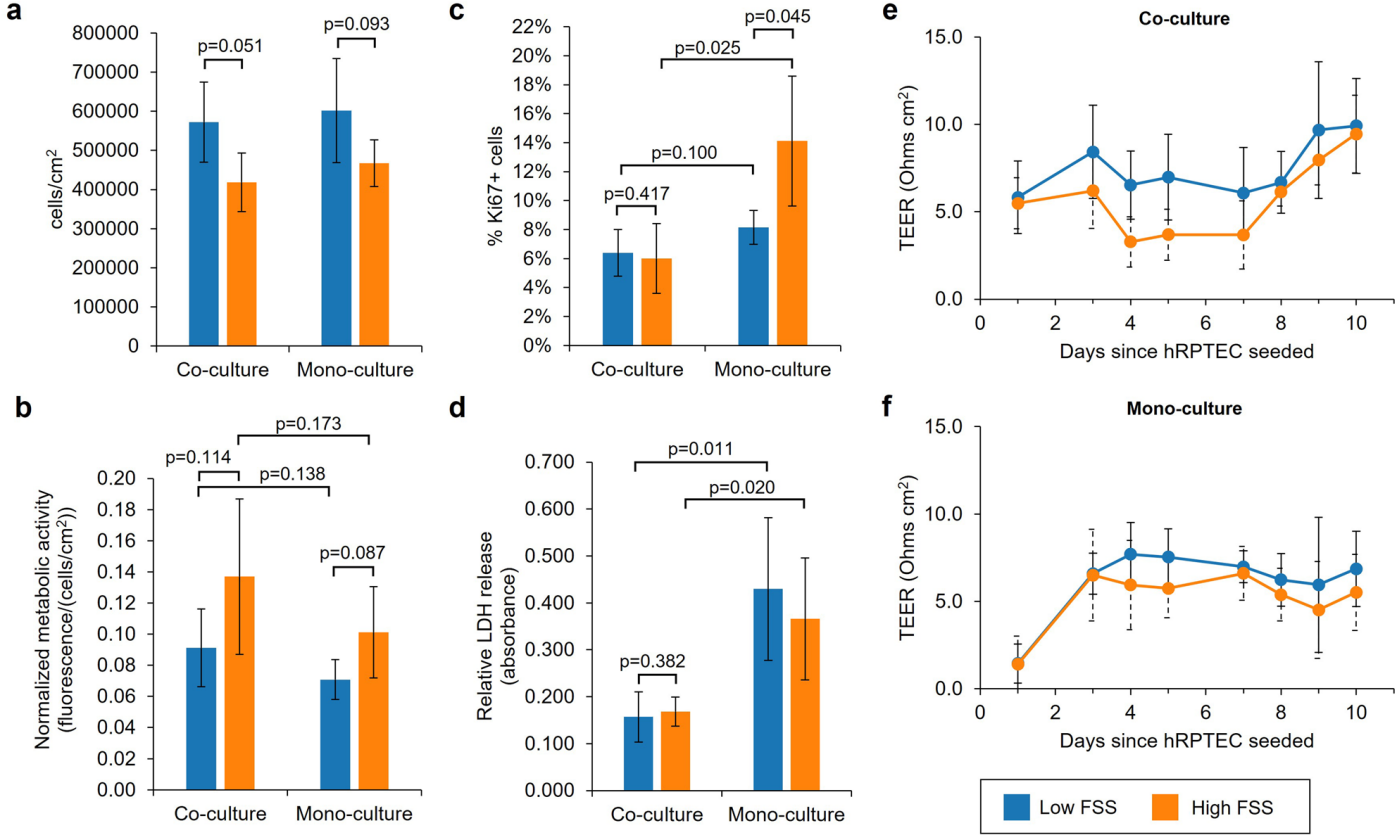
PREDICT96 facilitates hRPTEC characterization under multiple model conditions. (**a**) hRPTEC densities in the membrane overlap region are similar in co- and mono-culture conditions but tend to be higher in low FSS. (**b**) Relative bulk metabolic activity of hRPTEC normalized to cell density suggests higher metabolic rates among cultures in high FSS. (**c**) Percent of cells positive for proliferation marker Ki67 shows significantly higher proliferative activity among mono-cultures in high FSS (*p* = 0.025) and slightly higher rates among mono-cultures in low FSS (*p* = 0.100) compared to co-cultures, *n* = 3 images per device (see Methods). (**d**) Relative LDH release at baseline conditions highlights significantly greater cell death in mono-culture compared to co-culture in low FSS (*p* = 0.011) and in high FSS (*p* = 0.020). (**a**-**d**) *n* = 3 devices per condition, p values based on one-sided Students’ *t*-test, *α* = 0.05. (**e**) Average TEER of co-culture devices under baseline conditions showed minimal differences in barrier integrity after 10 days relative to FSS (*n* = 33 devices per FSS up to day 7, *n* = 6 on days 8–10). (**f**) Average TEER of mono-culture devices under baseline conditions showed negligible differences in barrier integrity relative to FSS and similar magnitude as co-culture devices (*n* = 9 devices per FSS up to day 7, *n* = 3 on days 8–10). *N* = 1 experimental replicate. Bars represent mean ± SD of biological replicates.

PREDICT96 enabled simultaneous evaluation of the kidney model under multiple conditions: with and without hMVEC co-culture and under two different levels of FSS. In mono-culture, hRPTEC reached confluency on the membrane (Supplementary Fig. S1) with a cell density that was comparable to co-culture by day 10 (Fig. 2a). However, hRPTEC in mono-culture failed to form the characteristic cobblestone morphology, appeared elongated, and many cells lacked primary cilia, instead showing strong cytoplasmic staining for acetylated tubulin (Supplementary Fig. S1). Consistent with the level of differentiation suggested by their morphology, hRPTEC tended to be less metabolically active in mono-culture than in co-culture, although this trend did not reach statistical significance (Fig. 2b). hRPTEC also tended to be more proliferative, based on Ki67 staining, on day nine in mono-culture, a trend that was statistically significant under high FSS (Fig. 2c, representative images in Supplementary Fig. S2). Notably, hRPTEC had significantly higher levels of baseline cell death, as measured by lactate dehydrogenase (LDH) release assay, in mono-culture compared to co-culture independent of FSS (Fig. 2d). Despite these differences, TEER was between 5 and 10 Ohm cm^2^ for both co-cultures and mono-cultures after ten days (Fig. 2e,f).

Compared to co-culture with hMVEC, the level of FSS had more modest effects on hRPTEC cultured in PREDICT96. Under high FSS (0.70 dyn/cm^2^) mimicking the PT physiological range^8^, hRPTEC in co-culture developed morphological characteristics of differentiated PT tissue including continuous tight junctions and primary cilia that protruded from the apical membrane (Fig. 1c-f). In regions of the tissues, tight junctions were noticeably biased towards the apical cell membrane, indicative of a polarized phenotype (Fig. 1e). hRPTEC exposed to low FSS (0.01 dyn/cm^2^) still developed characteristic features when in co-culture with hMVEC (Supplementary Fig. S3) but tended to reach higher densities on the membrane and be less metabolically active (Fig. 2a,b). Trends in mono-culture hRPTEC density and metabolic activity with respect to FSS were similar to those of hRPTEC in co-culture. Unlike co-culture conditions, however, hRPTEC in mono-culture were significantly more proliferative after exposure to high FSS compared to low FSS (Fig. 2c). The level of FSS did not appear to influence the level of baseline cell death in co-culture or mono-culture (Fig. 2d).

### PT model has dose-dependent cytotoxicity response to cisplatin

**Figure 3:**
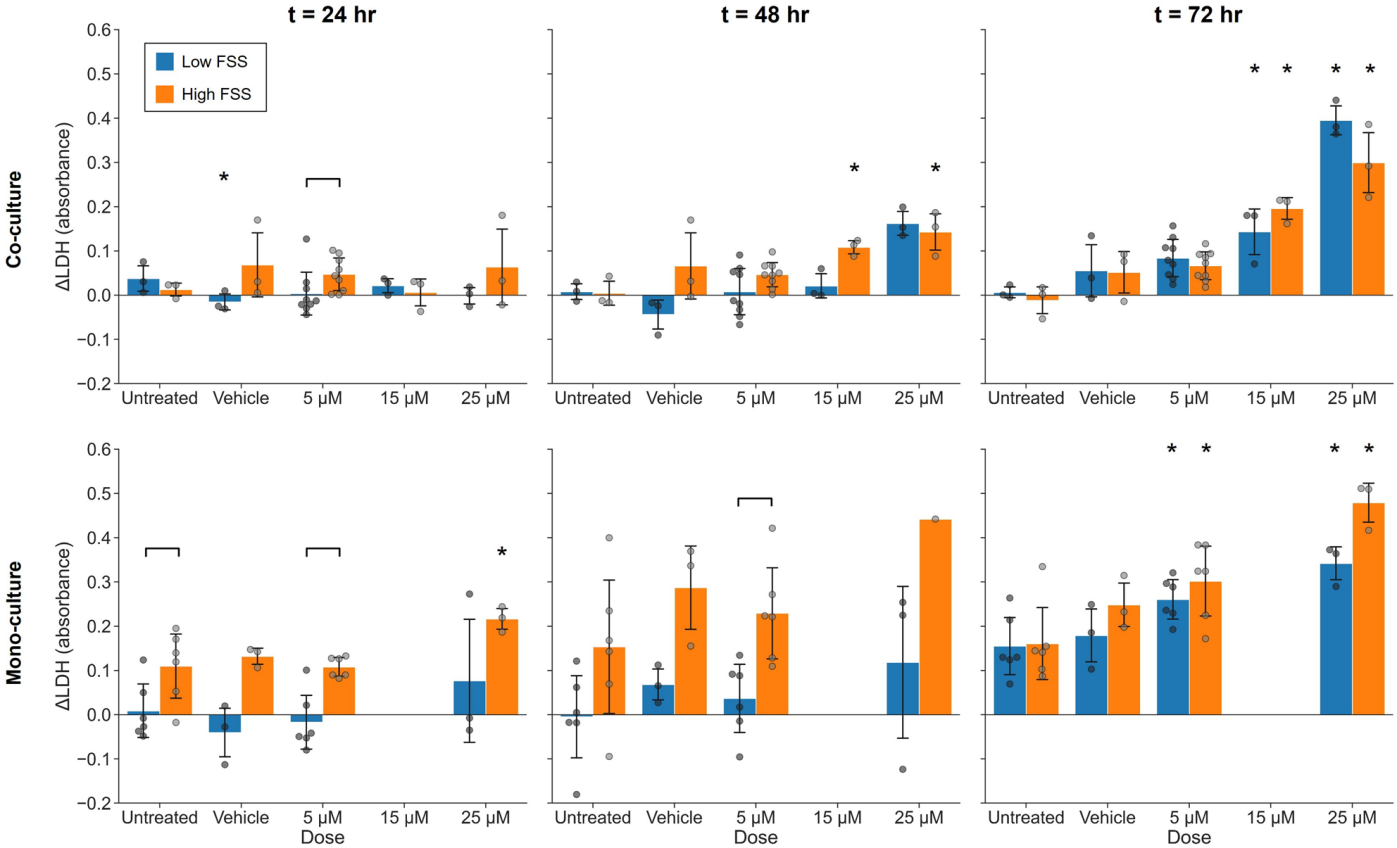
LDH release demonstrates dose-dependent cytotoxicity of cisplatin to the PT model. Plots show the net change in LDH release relative to baseline (*t* = 0 h) at various time points during cisplatin exposure for co-cultures (top row) and mono-cultures (bottom row). LDH release remained consistent over time in untreated co-cultures conditions but increased for untreated mono-cultures. LDH release increased with cisplatin concentration and duration of exposure. Bars represent mean ± SD of biological replicates. Co-culture: *n* = 3 devices per condition, except 5 μM cisplatin *n* = 9. Mono-culture: *n* = 6 for untreated and 5 μM, *n* = 3 for vehicle and 25 μM, 15 μM not evaluated. *N* = 1 experimental replicate. *p ≤ 0.05 Kruskal–Wallis with Dunn’s post-hoc test compared to corresponding untreated culture control at same time point. Brackets indicate significant comparisons (*p* ≤ 0.05) based on two-sided Mann–Whitney U-test.

To develop a model of drug-induced toxicity using the PREDICT96 kidney model, we exposed the model to a range of cisplatin concentrations (co-culture: 5, 15, 25 µM; mono-culture: 5, 25 µM) and assessed cytotoxicity using an LDH release assay. Higher doses of cisplatin were associated with increased LDH release compared to untreated controls (Fig. 3). A significant increase in LDH release was observed after 48 h of exposure to 15 or 25 µM cisplatin for co-cultures in high FSS conditions but was not observed until 72 h for co-cultures in low FSS. hRPTEC in untreated and vehicle-treated co-cultures showed relatively stable levels of LDH release over time. hMVEC in co-culture also exhibited a net increase in LDH which was significant within 24 h for the highest dose (Supplementary Fig. S4). In contrast, LDH release by hRPTEC in mono-culture tended to increase over time in untreated and vehicle conditions. LDH release increased significantly in mono-cultures under high FSS exposed to 25 µM cisplatin within 24 h. LDH release also appeared to increase in mono-cultures under low FSS after 48 h of exposure to 25 µM but, due to high variability, was not significant until 72 h. Mono-cultures also exhibited significantly increased LDH release after 72 h of exposure to 5 µM cisplatin, independent of FSS. 15 µM cisplatin was not evaluated for mono-culture tissues.

### Cisplatin induces loss of hRPTEC tight junction structure across FSS and culture conditions

**Figure 4:**
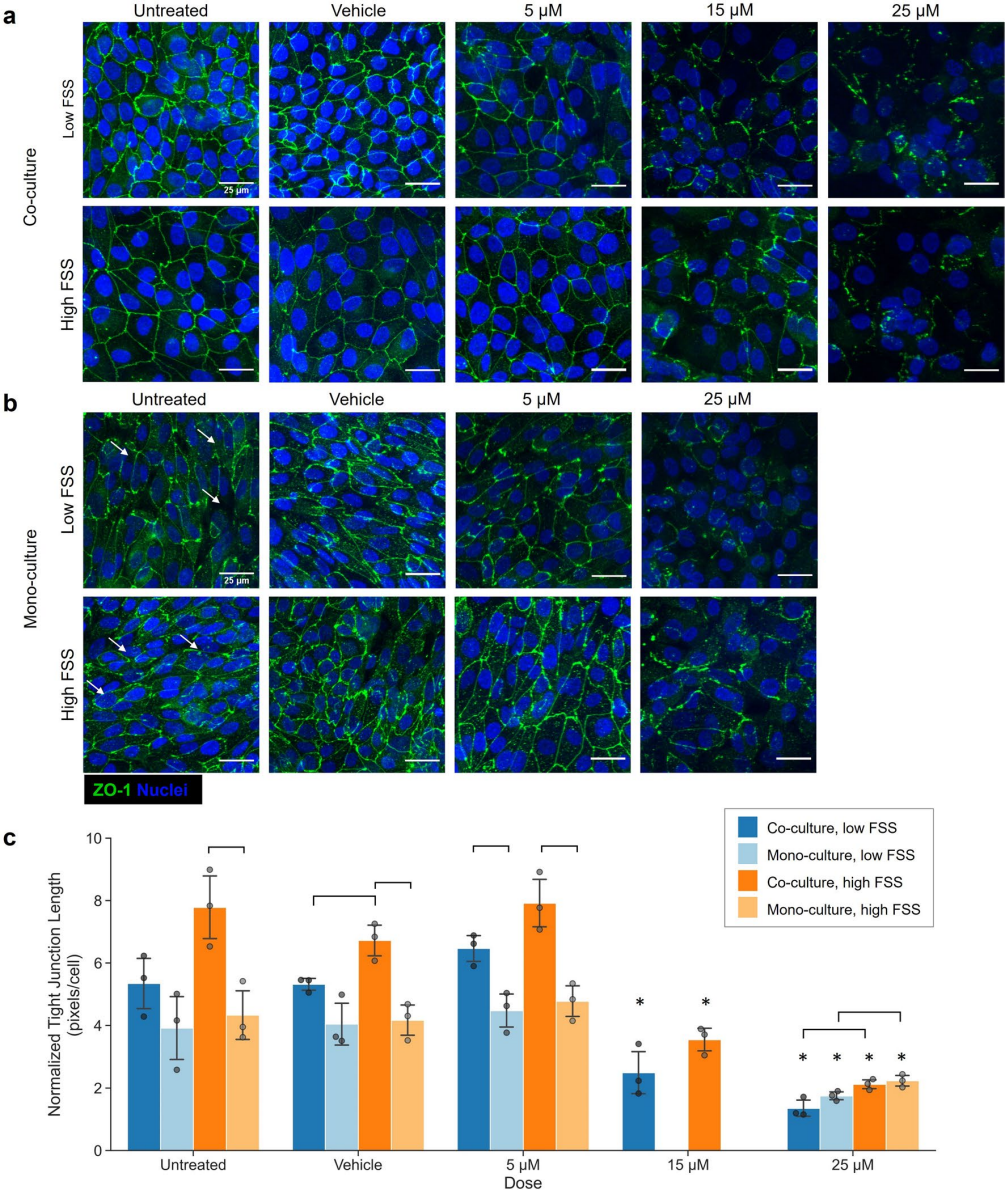
PT model displays dose-dependent loss of tight junctions after exposure to cisplatin. Representative images of hRPTEC tight junction morphology with respect to cisplatin concentration for (**a**) co-cultures and (**b**) mono-cultures after 72 h of exposure. Images are maximum intensity projections of confocal z-stacks of hRPTEC on the device membrane stained for ZO-1 (green) and nuclei (blue). White arrows indicate locations of missing tight junctions in untreated mono-cultures. (**c**) Normalized tight junction length (pixel length of junctions divided by total cells) corroborated that junctional coverage tended to be lower in mono-cultures than in co-cultures at a given FSS. Normalized tight junction length was reduced after exposure to 15 or 25 μM cisplatin. *N* = 3 devices per condition (biological replicates), where each data point is an average of three equally-sized fields of view per device. Bars represent mean ± SD of biological replicates. *p ≤ 0.05 single-factor ANOVA with Tukey’s post-hoc test relative to corresponding untreated control. Brackets indicate significant comparisons (*p* ≤ 0.05) based on two-sided Students’ *t*-test.

To determine whether changes in barrier function would be a meaningful metric of toxicity in the model, we evaluated PT tight junctions after 72 h of cisplatin exposure. Representative images of tight junction coverage are shown in Fig. 4. In co-culture, untreated and vehicle-treated hRPTEC exhibited well-connected junctional networks, whereas hRPTEC exposed to 15 and 25 μM cisplatin had noticeably fragmented tight junctions (Fig. 4a). Qualitative observations were corroborated by tight junction image quantification which indicated significant reductions in normalized tight junction length for hRPTEC exposed to 15 and 25 μM cisplatin (Fig. 4c). Cisplatin exposure was also associated with significantly reduced hRPTEC density in low FSS co-cultures (Supplementary Fig. S5). Consistent with prior observations, hRPTEC in mono-culture lacked complete tight junction networks in untreated conditions independent of FSS (Fig. 4b) and tended to have lower normalized tight junction length than hRPTEC in co-culture, though this trend was only statistically significant under high FSS (Fig. 4c). After exposure to 25 μM cisplatin, hRPTEC in mono-culture had noticeably fewer tight junctions and normalized tight junction length was significantly lower than untreated controls. Interestingly, normalized tight junction length was significantly lower after exposure to 25 μM cisplatin under low FSS compared to under high FSS both for co- and mono-culture conditions.

### TEER demonstrates dose-dependent changes in co-culture barrier function when exposed to cisplatin

**Figure 5:**
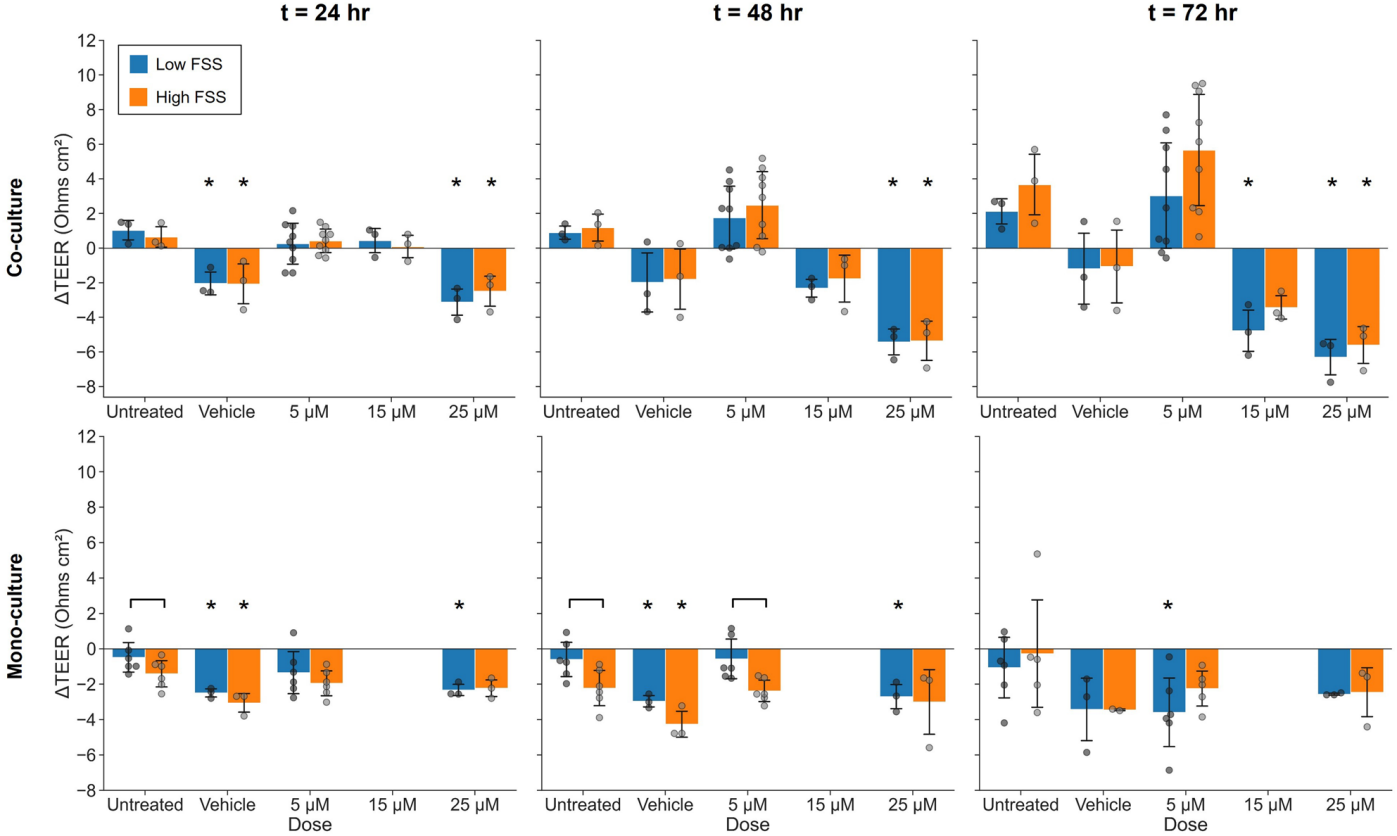
TEER sensing reveals dose-dependent changes in co-culture barrier function due to cisplatin exposure. Plots show the net change in TEER relative to baseline (*t* = 0 h) at various time points during cisplatin exposure of co-cultures (top row) and mono-cultures (bottom row). Co-culture TEER decreased with exposure to 15 and 25 μM and persistently increased with 5 μM, whereas mono-culture TEER generally decreased independent of exposure. Bars represent mean ± SD of biological replicates. Co-culture: *n* = 3 devices per condition, except 5 μM cisplatin *n* = 9. Mono-culture: *n* = 6 for untreated and 5 μM low FSS, *n* = 5 for untreated and 5 μM high FSS, *n* = 3 for vehicle and 25 μM (except vehicle high FSS *n* = 2), and 15 μM not evaluated. *N *= 1 experimental replicate. *p ≤ 0.05 Kruskal–Wallis with Dunn’s post-hoc test relative to corresponding untreated culture control at same time point. Brackets indicate significant comparisons (*p* ≤ 0.05) based on two-sided Mann–Whitney U-test.

TEER enabled rapid, non-invasive tracking of cisplatin-induced tissue damage in co-culture conditions (Fig. 5). Co-culture TEER decreased significantly within 24 h of exposure to 25 µM cisplatin and remained depressed over the 72 h under both high and low FSS. TEER decrease appeared later for co-cultures exposed to 15 µM cisplatin and was not significant compared to untreated controls until 72 h and only for tissues under low FSS. TEER decreased initially in the vehicle condition under both FSS conditions but was not significantly different from untreated controls beyond 24 h. Interestingly, co-culture TEER was increased in many devices after 72 h exposed to 5 µM cisplatin in both high and low FSS, though these changes were not statistically significant overall. In contrast, TEER tended to decrease for hRPTEC in mono-culture seemingly independent of cisplatin dose. Additionally, TEER did not decrease as significantly for mono-cultures as for co-cultures after exposure to 25 µM cisplatin under both FSS conditions (*p* = 0.05 one-sided Mann–Whitney, FSS-matched comparisons at 72 h). Mono-culture TEER under low FSS was significantly decreased after 72 h exposed to 5 µM cisplatin, consistent with elevated LDH

### Change in co-culture TEER aligns with tight junction expression and LDH release

**Figure 6:**
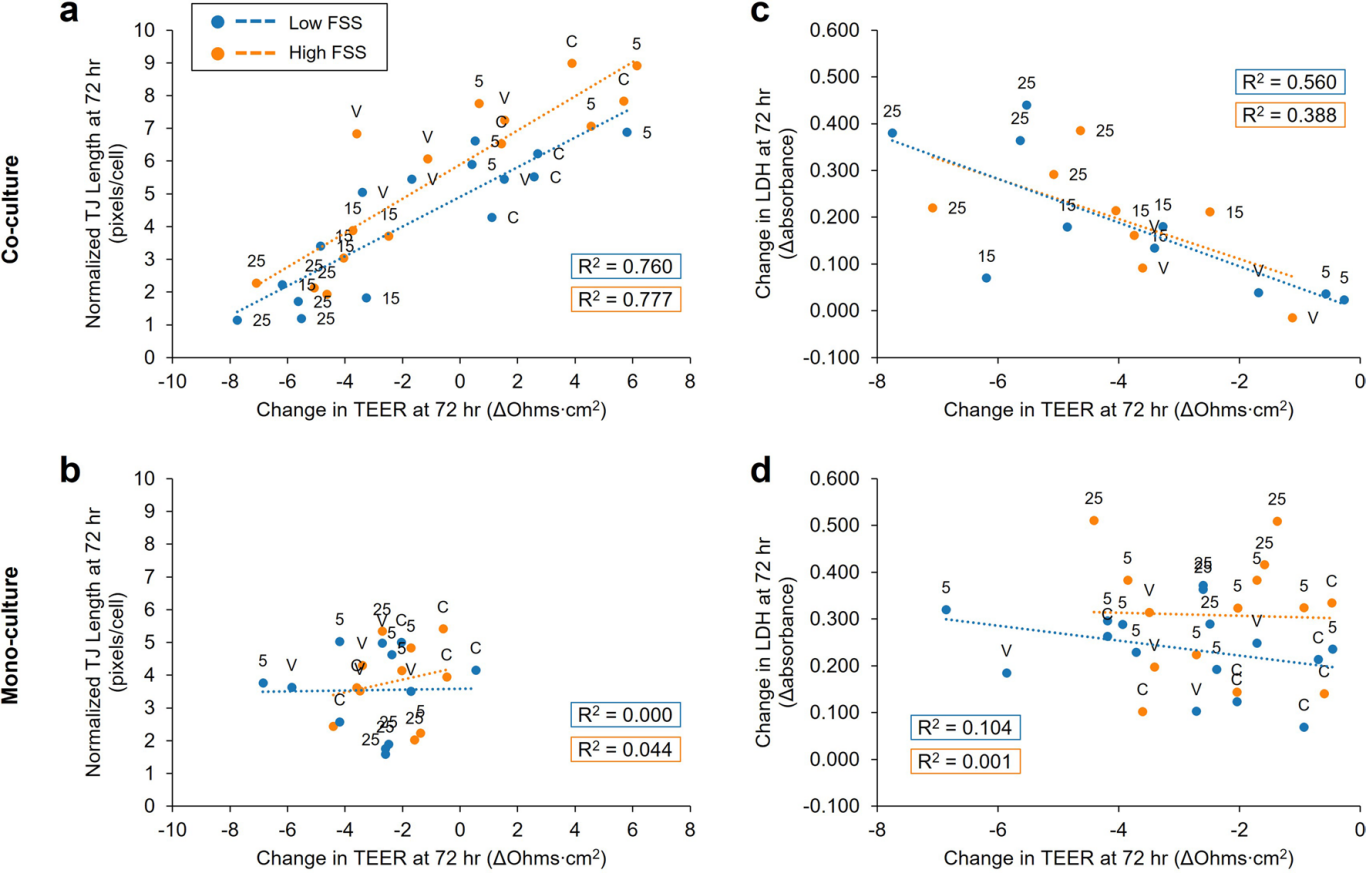
Change in co-culture TEER is an indicator of cisplatin-induced injury, corroborated by tight junction expression and LDH release. (**a**) Linear regression by ordinary least squares indicated a strong positive correlation between normalized tight junction (TJ) length (pixels/nuclei) and net change in TEER after 72 h for co-culture (Low FSS: R^2^ = 0.732, *p* < 0.001, *n* = 15; High FSS: R^2^ = 0.730, *p* < 0.001, *n* = 15). (**b**) No correlation between normalized tight junction length and TEER was evident for mono-culture (Low FSS: R^2^ = 0.001, *n* = 12; High FSS: R^2^ = 0.016, *n* = 11). (**c**) Linear regression also indicated a significant correlation between net change in LDH release and net change in TEER for co-culture low FSS devices that showed a decrease in barrier function (ΔTEER < 0) after 72 h (R^2^ = 0.511, *p* = 0.020, *n* = 10). Fewer co-culture high FSS devices showed a decrease in barrier function after 72 h, and a linear model between net changes in LDH and TEER for these devices did not reach statistical significance (R^2^ = 0.326, *p* = 0.139, *n* = 8). All data points represent biological replicates from *N* = 1 experimental replicate. (**d**) Changes in LDH and TEER were not correlated for mono-cultures under either low or high FSS (Low FSS: R^2^ = 0.169, *p* = 0.102, *n* = 17; High FSS: R^2^ ~ 0, *n* = 14). Data labels (**a**-**d**): (5,15,25) cisplatin concentration in μM, (C) untreated control, and (V) vehicle control.

To evaluate TEER relative to other metrics of kidney toxicity, we considered the relationships between change in TEER and hRPTEC tight junctions as well as net LDH release at 72 h. For hRPTEC in co-culture, change in TEER was well correlated with average normalized tight junction length under both low and high FSS (R^2 ^= 0.76 and 0.78 respectively, *p* < 0.001) (Fig. 6a). This was starkly juxtaposed with mono-culture for which there was no correlation between normalized tight junction length and change in TEER at 72 h (Fig. 6b). Change in TEER for co-cultures under low FSS that had a net reduction in TEER after 72 h (i.e. ΔTEER < 0) was also modestly correlated with net LDH release (R^2^ = 0.56, *p* = 0.013) (Fig. 6c). Co-cultures under high FSS showed a similar trend, but fewer co-cultures under high FSS exhibited a net reduction in TEER and the linear model did not reach statistical significance (R^2^ = 0.39, *p* = 0.099). In contrast, change in TEER for mono-cultures was not significantly correlated with LDH release for either FSS condition (Fig. 6d).

## Discussion

Microfluidic tissue culture systems offer significant improvements over existing in vitro models used in preclinical toxicological screens by enabling complex culture conditions, such as fluid flow and co-culture, to better mimic the native microenvironment. However, the flexibility to rapidly evaluate changes in kidney function in response to drugs with temporal resolution across multiple culture conditions is still needed to achieve a more comprehensive understanding of nephrotoxicity risks during preclinical studies^3^. TEER sensing is a well-established, non-invasive technique with rapid measurement potential but has only recently been implemented in high-throughput microfluidic in vitro models^21,29^. Others have demonstrated the potential to use TEER sensing for PT injury detection^16,25,30^, but these models did not utilize primary cells with barrier properties similar to the native PT and incorporate endothelial co-culture. Moreover, these studies were not performed in culture systems that provide user-controlled, multi-flow capability in an industry-standard format readily scaled for high-throughput analyses. Our kidney model incorporates primary PT cells co-cultured with primary microvascular endothelial cells and was developed in the high-throughput, flow-enabled PREDICT96 platform with rapid TEER sensing capability, providing an ideal context in which to screen culture conditions and evaluate TEER-based detection of drug-induced toxicity.

While models utilizing a single cell type reduce operational complexity, our study shows that co-culture with hMVEC strongly influences hRPTEC tissue structure in PREDICT96. When co-cultured with hMVEC, hRPTEC exhibited continuous tight junctions and robust primary cilia expression, indicative of a differentiated PT phenotype (Fig. 1). In contrast, hRPTEC in mono-culture showed more gaps in tight junction coverage, less abundant primary cilia, and had more elongated nuclei (Supplementary Fig. S1). Mono-cultured hRPTEC were more likely to show expression of the proliferative marker Ki67, especially under high FSS, and to undergo cell death after almost a week in unchanging conditions. Since there were negligible differences in hRPTEC density between mono-culture and co-culture (when exposed to the same FSS), we hypothesize that mono-culture was associated with higher rates of cell turnover as confocal imaging suggested abundant cell extrusion^31^ (Supplementary Fig. S1). Moreover, hRPTEC from a different primary donor also showed more robust tight junction formation in co-culture conditions independent of FSS in PREDICT96 (Supplementary Fig. S6). Together, these observations suggest that hMVEC co-culture enhanced hRPTEC tissue structure formation and led to more stable monolayers, consistent with previous in vitro studies^13,14^.

The native PT is a highly permeable tissue, and primary PT cells cultured in vitro exhibit TEER values orders of magnitude lower than other barrier forming tissues^28^. hRPTEC cultured in PREDICT96, with the current device membrane specifications (see Methods), formed permeable barriers with TEER values between 5 and 10 Ohms cm^2^ in both co-culture and mono-culture conditions (Fig. 2). These values are similar to the average TEER of 6.4 Ohms cm^2^ recently reported for primary RPTEC cultured in a membrane-free microfluidic platform^21^. Despite the similar TEER values of co- and mono-cultures, we observed notable differences in hRPTEC tight junction coverage between culture types. This seeming disagreement between TEER and tight junctions could be explained by differences in tight junction structure and protein composition as well as cell-substrate adhesion, since the combined effects of these factors impact the value of TEER^32–35^. Microvascular endothelial cells also express tight junctions, so their potential contribution to TEER in the co-culture model cannot be discounted.

Previous research has demonstrated substantial improvements in PT cell viability and differentiation when exposed to FSS stimulation in vitro compared to static culture^6,7,24,36,37^. Although studies have explored the effects of a wide range of FSS intensities, there remains a lack of consensus on the precise level of stimulation that is ideal for PT tissues in vitro^38^. Moreover, physiological FSS is variable due to differences in tubule diameter, tortuosity, and rates of reabsorption, and diseases contribute to further irregularity^8^. Since it is not clear whether an ideal FSS exists for toxicity screening assays, it is advantageous for culture platforms to enable model characterization under multiple flow conditions. In our study, the two levels of FSS intensity applied did not have a significant impact on hRPTEC phenotype, but co-cultured hRPTEC exposed to low FSS (0.01 dyn/cm^2^) tended to form denser monolayers with lower metabolic activity per cell than those exposed to high FSS (0.70 dyn/cm^2^). A similar trend was observed for hRPTEC in mono-culture with the additional effect that high FSS stimulated greater proliferative activity after a week in culture. These results suggest that FSS impacted cell and monolayer homeostasis. Consistent with our observations, Ross et al. recently reported differential expression of PT metabolism associated genes in RPTEC/TERT1 exposed to varying levels of FSS in the range of 0.1 to 0.5 dyn/cm^2^^39^. Thus, while viability and tissue barrier function may be minimally affected, levels of FSS may have prominent effects on PT metabolism, an important factor to consider since many drugs undergo biotransformation in the kidney^40^. It is also notable that use of the PREDICT96 platform enabled us to isolate the effect of FSS exposure on hRPTEC by modulating fluid flow in the hRPTEC channel independently of flow in the hMVEC channel. Since hMVEC are also FSS-responsive^41^, uniform flow modulation could confound the direct effects of FSS on hRPTEC. The capability to screen multiple flow conditions with channel-specific control could help clarify toxicity differences between individuals or those associated with disease.

Cisplatin is a highly nephrotoxic anticancer agent which has been widely applied in the development of in vitro models for kidney toxicity screening due to its well-characterized, dose-dependent toxicity^42,43^. We employed a cisplatin-induced toxicity model to evaluate the feasibility of TEER-based detection of drug-induced injury in our microfluidic kidney model. Clinical dosing regimens for cisplatin vary significantly in concentration (15 to 100 mg/m^2^) and frequency (five consecutive days to once every three weeks) depending on cancer type and patient-specific toxicity risk^44,45^. The concentrations of cisplatin applied in this study are similar to peak blood concentrations observed during a typical infusion^44,45^ and on the low end of typical concentrations used for in vitro studies^16,22,26,46,47^.

The LDH release assay has been widely applied for preclinical toxicology and measures cell death based on the detection of intracellular LDH enzyme which is released into the culture medium by ruptured cells^48^. We performed LDH assays to evaluate the cytotoxic effects of cisplatin on our kidney model and provide a comparison for TEER-based detection of injury (Fig. 3). Cell death in co-cultured hRPTEC increased with cisplatin concentration and exposure time. Cell death was significantly elevated in 15 and 25 µM cisplatin within 48 h with high FSS but was not elevated until 72 h with low FSS, perhaps related to greater metabolic activity per cell under high FSS previously noted. For mono-cultures, 25 µM cisplatin increased cell death within 24 h under high FSS, and 5 µM cisplatin increased cell death within 72 h independent of FSS. However, hRPTEC death in mono-culture increased more significantly than in co-culture over the 72-h period in untreated conditions, highlighting the potential for greater sensitivity with a co-culture-based toxicity assay (Mann–Whitney U-test: low FSS *p* = 0.028, high FSS *p* = 0.024) hMVEC also exhibit cisplatin sensitivity^49^, and we observed increased cell death in the hMVEC channel of co-cultures exposed to 15 and 25 µM doses. Our observations have shown that hMVEC crosstalk influences hRPTEC phenotype and, as such, it may also play a role in modulating hRPTEC response to injury. While clarifying the mechanisms of this relationship was beyond the scope of this work, we believe the data nonetheless demonstrate the utility of the co-culture model while highlighting the potential for future work.

Tight junction coverage, quantified as normalized tight junction length, was another metric of cisplatin toxicity applied to evaluate the model (Fig. 4). Co-cultures exposed to 15 and 25 µM cisplatin and mono-cultures exposed to 25 µM cisplatin showed significant tight junction damage after 72 h and had significantly lower normalized tight junction length compared to untreated controls. These results agree strongly with previous work by Nieskens et al. who reported tight junction disruption and reduced cell count in a primary PT model exposed to 25 µM cisplatin for 72 h^47^. Interestingly, normalized tight junction length was significantly lower after exposure to 25 uM under low FSS compared to under high FSS in both culture conditions, suggesting a potential role for FSS in the model’s response to injury.

Consistent with tight junction expression, TEER sensing revealed changes in the barrier function of co-cultures during cisplatin exposure (Fig. 5). Reduced TEER was significant within 24 h for co-cultures exposed to 25 µM cisplatin under both FSS conditions. These changes were detected up to 48 h before significant changes in LDH release under low FSS and up to 24 h before under high FSS. Barrier function of co-cultures exposed to 15 µM cisplatin showed a decreasing trend but was only reduced to a significant degree under low FSS after 72 h. Interestingly, TEER of many co-cultures persistently increased with exposure to 5 µM cisplatin. Increased barrier function in response to low concentrations of cisplatin has been reported by other authors ^17,26,50^. Vormann et al. reported a permeability decrease in RPTEC treated with 5 µM cisplatin after 24 h^17^, and Wilmes et al. observed TEER increases with RPTEC/TERT1 after exposure to 0.5 µM and 2 µM cisplatin^26^. While we did not observe significant differences in normalized tight junction length among co-cultures exposed to 5 µM cisplatin, changes in junction composition that reduced paracellular permeability may have driven the observed increases in TEER^50^. Junctional remodeling may represent an early stress response that precedes the lethal effects of cisplatin exposure, highlighting an advantage of TEER sensing for investigations of toxicity. Additionally, this increasing trend was not observed in mono-cultures exposed to 5 µM cisplatin, suggesting a role for hMVEC crosstalk in modulating hRPTEC response to toxic compounds. For mono-cultures, TEER decreased independent of cisplatin exposure and did not drop as substantially as in co-cultures when exposed to 25 µM cisplatin. This may be due to the less differentiated phenotype of hRPTEC in mono-culture, and future experiments could help clarify whether this lead to reduced cisplatin uptake^49^. Cisplatin-dosed hRPTEC from a different primary donor demonstrated a similar trend with prominent dose-dependent changes in TEER observed when co-cultured with hMVEC and minimal changes when cultured alone, further corroborating the utility of TEER when applied to co-culture PT models. These results demonstrate that it is feasible to detect toxicity-induced changes in barrier function in a microfluidic PT co-culture model that recapitulates the leakiness of the native PT and that TEER is potentially an earlier indicator of tissue response than cell death.

The high-throughput PREDICT96 TEER system generates readouts for 96 devices in less than 8 min, a significant improvement over conventional toxicity assays like LDH release or barrier metrics like tight junction expression. We found that normalized tight junction length was well correlated with change in TEER at 72 h for co-cultures independent of FSS exposure (Fig. 6). Greater reductions in TEER were generally associated with greater increases in LDH release for co-cultures, consistent with a loss of barrier function due to cell death, and the linear trend was significant for co-cultures under low FSS. Since mono-culture TEER changed similarly across treatment groups, it was not a clear predictor of tight junctions or LDH release. These results demonstrate that TEER is a relevant metric of cisplatin toxicity in the co-culture model.

The results of this study highlight the potential of microfluidic PT models, through the integration of high-throughput non-invasive sensing technologies, to generate rapid predictions of drug-induced toxicity informed by kidney-specific readouts. Future investigations of TEER-based toxicity detection in primary cell kidney models will need to involve compounds from a range of drug classes in order to determine the broader applicability to preclinical screening. In addition to efficient readouts of barrier function, TEER sensing technology is conducive to multiplexing with other PT toxicity indicators, such as oxygen consumption and injury marker secretion, which could enable the generation of multi-parametric datasets with more predictive power. Broader application of rapid TEER sensing technology could also improve understanding of tight junction dynamics in a variety of biological contexts including diseases of the PT, such as polycystic kidney disease^51^, or in other tissue types such as lung^52^, vascular^53^, and liver^54^, which have been previously modeled in PREDICT96.

## Conclusion

TEER sensing provides a functional readout for kidney tissues and can be scaled to meet the needs of high-throughput screening with microfluidic models. We implemented the PREDICT96 rapid TEER system to evaluate the feasibility of using TEER sensing for toxicity detection in co-culture and mono-culture PT models exposed to two different levels of FSS. We demonstrated that TEER was an informative metric of cisplatin toxicity for hRPTEC-hMVEC co-cultures but not for hRPTEC in mono-culture. Changes in co-culture TEER correlated with hRPTEC tight junction expression and aligned with trends demonstrated by a standard cytotoxicity assay. Notably, TEER sensing revealed a decrease in co-culture barrier function due to cisplatin that occurred prior to significant increases in cell death. FSS appeared to influence hRPTEC metabolism as well as the onset of adverse effects, reinforcing the value of platforms that provide the flexibility to simultaneously test multiple flow rates. TEER sensing shows promise as a tool for assaying kidney toxicity in high-throughput microfluidic models and has potential to contribute to more predictive preclinical screens.

## Methods

### Cell culture

Primary human renal proximal tubule cells (hRPTEC) purchased from ScienCell Research Laboratories (4100) were utilized for experiments at passage 6. Cells were cultured in hRPTEC growth medium consisting of Dulbecco's Modified Eagle Medium: Nutrient Mixture F-12 supplemented with 10 ng/mL human epidermal growth factor, 5 µg/mL insulin from bovine pancreas, 0.5 µg/mL hydrocortisone, 0.5 µg/mL (-)-epinephrine, 6.5 ng/mL 3,3′,5-Triiodo-L-thyronine sodium salt, 10 µg/mL human transferrin, and 0.5% fetal bovine serum. Primary human dermal microvascular endothelial cells (hMVEC) purchased from Lonza (CC-2543) were expanded through passage 6 in flasks coated with 1 µg/cm^2^ human plasma fibronectin and cultured in the vendor-recommended growth medium, EGM-2MV, excluding gentamicin-amphotericin B.

### PREDICT96 plate & pump preparation

PREDICT96 microfluidic culture plates and microfluidic pumps were fabricated as previously described^29^. Plates contained a microporous polycarbonate track-etched (PCTE) membrane with a nominal pore diameter of 1 µm, thickness of 10 µm, and 16% open area. PREDICT96 plates and pumps were sterilized by ethylene oxide exposure for 12 h followed by at least 48 h of degassing in a vacuum chamber. Plates were treated with oxygen plasma for 60 s to promote hydrophilicity and rinsed three times with phosphate buffered saline (PBS) before filling with media for blank resistance measurement. Pumps were primed and calibrated as previously described^29^. The average stroke volume of the micropumps (1.06 µL) was used to calculate the stroke frequency (flow rate divided by stroke volume) needed to achieve the desired flow rates.

### PREDICT96 plate seeding & culture

Before the first cell type seeding, devices were coated with 60 µg/mL collagen from human placenta in 0.25% acetic acid by incubating for 60 min at room temperature (RT) followed by two rinses with PBS. hMVEC were seeded in the top channel of each device two days prior to seeding hRPTEC. Both cell types were seeded by the gravity method^29^ which involved quickly aspirating the media from the top channel inlet wells and replacing it with 30 µL of ~ 1 million/mL cell suspension. For hRPTEC seeding in the bottom channel, the plate was flipped face-down immediately after loading cell suspension to allow cells to attach to the bottom side of the membrane. The plate was incubated at 37 °C for at least 1 h in static conditions to allow cells to settle before the first media change and flow initiation at 1µL/min in both channels. On subsequent days, flow was increased to ‘high FSS’ in a portion of the devices according to the regime shown in Supplementary Fig. S7. Flow ramping consisted of two step increases in pumping rate and was performed to minimize the disturbance to the tissue layers. ‘Low FSS’ reflects 0.01 dyn/cm^2^ in the hRPTEC channel and 0.70 dyn/cm^2^ in the hMVEC channel, and ‘high FSS’ reflects 0.70 dyn/cm^2^ in both channels. The entire volume of media in each channel (120 µL) was refreshed daily.

### Transepithelial electrical resistance (TEER) measurement

TEER was measured daily prior to media changes. Measurements were taken in a biosafety cabinet after stopping fluid flow. A CultureTemp 37 °C warming plate (SP Bel-Art) was used to minimizing cooling of the culture medium during measurement. Prior to collagen coating and cell seeding, the blank resistance of each device was measured (typically ~ 3000 Ohms). Our prior analysis has shown that collagen coating does not substantially alter device resistance (− 1.8 ± 3.4%). Tissue TEER (Ohms cm^2^) for each device was calculated by subtracting the blank resistance from the resistance measured each day and multiplying by the surface area of the membrane in the overlap region of the device (~ 0.037 cm^2^). Measurements were discarded if bubbles were present in the channels. Several devices on each plate were not seeded and the blank resistance of these devices was tracked daily over the culture period. The average deviation in resistance of the blank devices from the original set of measurements was used to adjust the tissue TEER values each day in order to correct for variation in environmental factors (e.g. temperature, pH). TEER values shown in Fig. 2 were measured with a prototype electrode array and data were collected by reading resistance values directly from the EVOM2 (WPI Instruments) display. TEER data presented in Figs. 5 and 6 were collected with the PREDICT96 rapid TEER system, previously described^29^, which integrates with the EVOM2 to provide automated TEER measurement acquisition for all 96 devices. The rapid TEER system has theoretical limit of detection of ~ 0.22 Ohm·cm^2^ calculated as follows. The EVOM outputs a voltage signal ranging from − 12 to + 12 V where 1 mV corresponds to 1 Ohm. This signal is transmitted to the microcontroller analog-to-digital converter which has 12-bit resolution (24 V/2^12^ = 5.9 mV or 5.9 Ohms). Scaled to the area of the membrane in the microfluidic device (multiply by 0.037 cm^2^), this yields a TEER measurement resolution of ~ 0.22 Ohm·cm^2^. Resistance readouts were validated against a range of known resistors (Supplementary Fig. S8). Measurement precision is estimated at ~ 0.10 Ohm·cm^2^ based on the standard deviation of 15 replicate reads which was relatively consistent at across resistances for a minimum of 25 samples per read. 25 consecutive measurements were acquired for each device, and the median voltage was converted to resistance based on Ohm’s law in order to calculate tissue TEER as described above. The median voltage was used instead of the mean to minimize the effect of random noise and to prevent extrapolation to sub-resolution values. Measurement acquisition for all 96 devices took less than 8 min. All error bars for TEER data reflect the standard deviation of biological replicates.

### Experimental design

A timeline of the experiment is provided in Supplementary Fig. S7. Toxicity testing was performed using a single PREDICT96 culture plate, and dose groups were spatially randomized within blocked sections of co-culture and mono-culture to minimize the impact of positioning on tissue responses. Most groups consisted of *n* = 3 devices, but additional replicates were included for groups treated with 5 µM cisplatin and for untreated mono-cultures for greater statistical power.

### Cisplatin dosing

Cisplatin was dissolved in 0.09% (w/v) sodium chloride (aqueous) vehicle at a concentration of 0.5 mg/mL. The cisplatin stock solution was diluted to the working concentration in culture medium. The vehicle control contained an equivalent concentration of sodium chloride solution as the highest dose of cisplatin. Tissues were dosed via the top channel of each device (containing hMVEC in co-culture devices and empty in mono-culture devices) since basolateral membrane exposure was previously found to enhance the toxicity of cisplatin to hRPTEC in a microfluidic model^47^. Cisplatin was refreshed every 24 h immediately following TEER measurement, and untreated control devices received media changes at the same intervals.

### Lactate dehydrogenase (LDH) release assay

Media was extracted from each device channel at 24-h intervals during cisplatin exposure (i.e. *t* = 0, *t* = 24 h, *t* = 48 h, *t* = 72 h). Relative LDH content was measured immediately after collection using a CyQUANT LDH Cytotoxicity Assay Kit (Invitrogen, C20300). Samples were mixed on a shaker plate for at least 5 min and then 15 µL in triplicate was transferred to clear-bottom black-walled 384-well assay plates. 15 µL of reaction mixture was added to each well and plates were incubated at room temperature for 30 min protected from light. 15 µL of stop solution was added to each well and plates were briefly mixed on a shaker plate. Plates were centrifuged to eliminate bubbles before reading. Absorbance at 490 nm and 680 nm (background) was measured on a microplate reader. Relative LDH content was quantified by subtracting the background absorbance and the average absorbance of fresh medium and then averaging triplicate wells. Changes in LDH release were calculated as the difference between LDH at a given time point and the baseline on day 6 (*t* = 0).

### Metabolic activity assay

Metabolic activity assays were performed on the last day of culture immediately prior to tissue fixation. PrestoBlue™ HS cell viability reagent (Thermo Fisher, P50200) was diluted 1:20 in warm hRPTEC medium and added to each channel of each PREDICT96 device. Devices were incubated at 37 °C for 30 min with pumping at 10 µL/min in both the top and bottom channels. Media was collected from each channel and fluorescence was measured on a microplate reader with an excitation/emission wavelengths of 560/590 nm.

### Immunofluorescence staining & microscopy

Tissues were fixed by first quickly rinsing all channels with cold PBS and then incubating with 4% paraformaldehyde in PBS for 10 min at room temperature. Tissues were rinsed with PBS three times for 5 min each. Tissues were permeabilized with 0.1% Triton X-100 in PBS for 10 min at room temperature followed by three additional PBS rinses. Tissues were blocked in 3% normal goat serum (NGS) in PBS for 1 h at room temperature. Mouse and rabbit primary antibodies diluted in NGS buffer, and tissues were incubated in the solution overnight at 4 °C. Tissues were rinsed three times with PBS to remove unbound primary antibody. Goat anti-mouse IgG H&L Alexa Fluor 488 and goat anti-rabbit IgG H&L Alexa Fluor 568 secondary antibodies were each diluted 1:250 in NGS buffer. Nuclei and filamentous actin were co-stained using Hoechst 33342 (1:250) and Phalloidin-iFluor 633 (1:1000). Tissues were incubated with secondary antibodies and co-stains for 1-3 h at room temperature and then rinsed twice with PBS. Images were acquired with a scanning confocal microscope (Zeiss LSM700) equipped with 405 nm, 488 nm, 555 nm, and 637 nm lasers and 10 × air and 40 × water immersion objectives.

### Ki67 quantification

A pipeline was developed in Cell Profiler 3.0^55^ to quantify the proportion of cells positive for Ki67. Briefly, nuclei objects were identified based on Hoechst staining and then used as a mask to evaluate Ki67 staining intensity on a cell-by-cell basis. The number of cells expressing Ki67 above a specific intensity threshold divided by the total number of cells was taken as Ki67 positivity (%). A constant intensity threshold was applied to all images. The dataset consisted of three images in the overlap region per device and three devices per condition.

### Tight junction image quantification

Confocal z-stack images of nuclei- and ZO-1-stained tissues (blue and green channels, respectively) were acquired with a 40 × water immersion objective. Images were only collected in the overlap region of the device. Summed intensity projections of the nuclei channel were created in Fiji^56^ and analyzed with the StarDist2D plugin^57^ to estimate cell count. Maximum intensity projections of the ZO-1 channel were created, filtered using Gaussian blur (two-pixel radius), and analyzed with the Ridge Detection plugin^58^ in Fiji using the same parameters for all images. For each image, the plugin identified ridge-like structures at least 10 pixels in length (based on intensity gradients) and returned a binary image of the identified ridges reduced to single-pixel width (Supplementary Fig. S9). The total number of white pixels per image (‘total tight junction length’) was divided by the total nuclei in the image (‘cell count’) to calculate the normalized tight junction length. The results from three images per device were averaged, and three devices per group were evaluated.

### Statistical analysis & plotting

Experimental data were analyzed and plotted using Microsoft Excel and Python 3.0 with statistical analysis and plotting packages^59–61^. All bar plots indicate mean ± standard deviation of biological replicates. Since dose groups consisted of a variable number of replicates, the Kruskal–Wallis test (ANOVA by ranks) was implemented to evaluate comparisons relative to controls. A normality assessment was also performed for each dataset, and parametric tests were only applied for datasets that passed the Shapiro–Wilk test (Supplementary Fig. S10). A statistical significance threshold of *p* ≤ 0.05 was adopted for all tests.

### Ethical approval

No ethics approval was required for this work.

## Supplementary Information

Supplementary Information.

## Data Availability

The datasets generated during and/or analyzed during the current study are available from the corresponding author upon reasonable request.
