# Supplementary material for: Evaluation of rapid transepithelial electrical resistance (TEER) measurement as a metric of kidney toxicity in a high-throughput microfluidic culture system
Source: Sci Rep. 2022 Aug 1;12:13182. doi: 10.1038/s41598-022-16590-9 (PMC9343646; doi:10.1038/s41598-022-16590-9)
Supplement: Supplementary file 1 — Supplementary Information. [file 41598_2022_16590_MOESM1_ESM.pdf]

Supplementary Information

Evaluation of rapid transepithelial electrical resistance (TEER) measurement as a metric of kidney toxicity in a high-throughput microfluidic culture system

Erin M. Shaughnessey[1,2], Samuel H. Kann[1,3], Hesham Azizgolshani[1], Lauren D. Black III[2], Joseph L. Charest[1], & Else M. Vedula[1]*

[1]The Charles Stark Draper Laboratory Inc., 555 Technology Square, Cambridge, MA 02139, USA

[2]Department of Biomedical Engineering, Tufts University, 4 Colby Street, Medford, MA 02155

[3]Department of Mechanical Engineering, Boston University, 110 Cummington Mall, Boston, MA 02215

*Corresponding author, evedula@draper.com

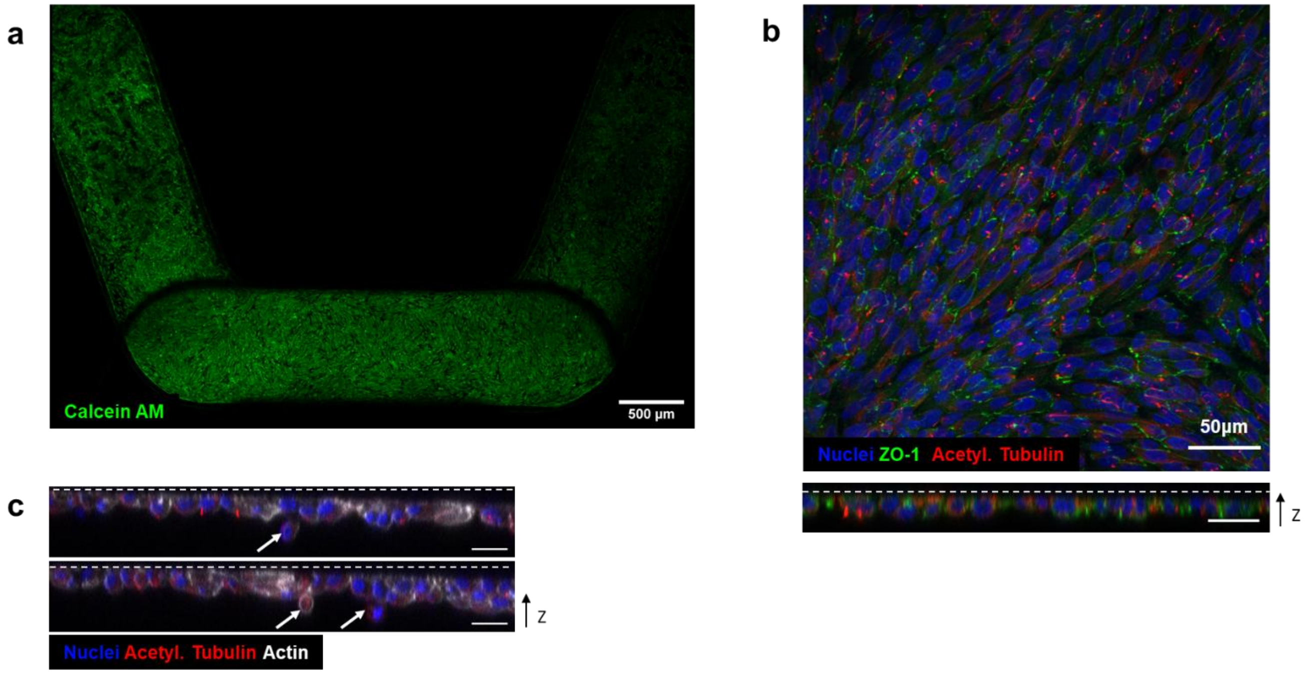

Supplementary Figure S1. hRPTEC morphology in mono-culture under high FSS. (**a**) hRPTEC in mono-culture live stained with Calcein AM after three days under high FSS (0.70 dyn/cm^2) in top channel of PREDICT96 device. (**b**) hRPTEC expression of tight junctions (ZO-1; green) and primary cilia (acetylated tubulin; red) after nine days in PREDICT96. (**c**) Orthogonal views of cells extruding from the apical surface of the hRPTEC monolayer. (**b**-**c**): Scale bars = 25 µm for orthogonal views. White dotted line indicates location of membrane in orthogonal views.

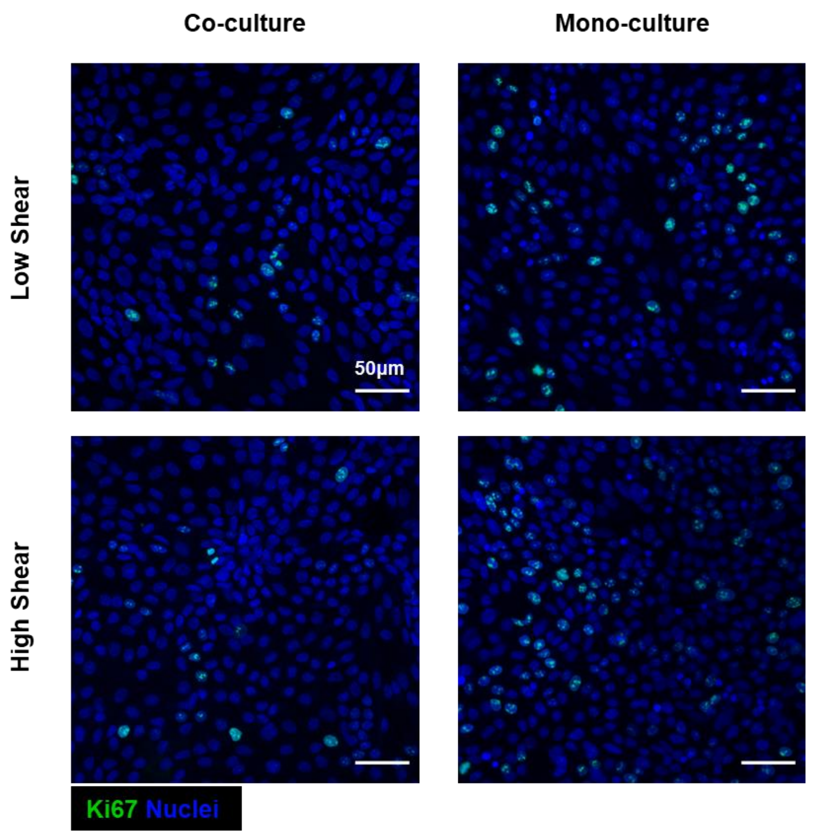

Supplementary Figure S2. hRPTEC Ki67 expression under different conditions. Representative images of hRPTEC Ki67 expression (green) in co-culture with hMVEC or mono-culture and exposed to either low (0.01 dyn/cm^2) or high (0.70 dyn/cm^2) FSS. Scale bars = 50 µm.

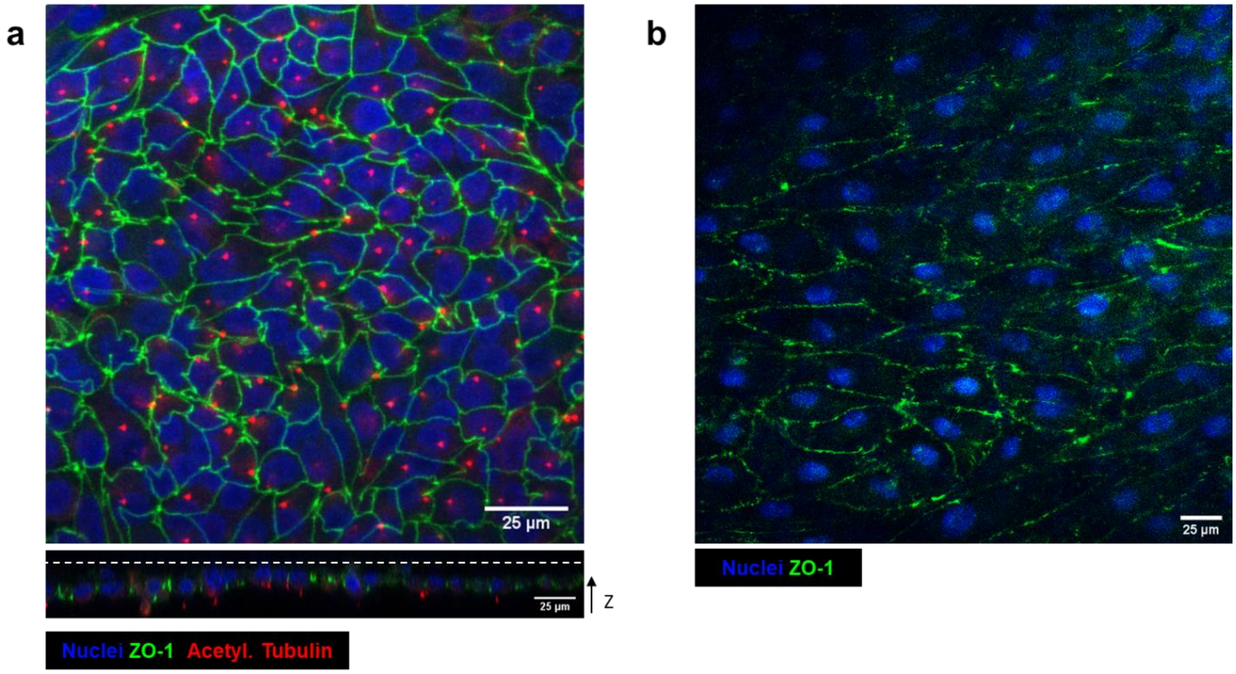

Supplementary Figure S3. Co-culture under low FSS. (**a**) Representative images of hRPTEC tight junctions (ZO-1; green) and primary cilia expression (acetylated tubulin; red) in co-culture after ten days in low FSS (0.01 dyn/cm^2). White dotted line indicates location of the membrane in orthogonal view. (**b**) hMVEC under high FSS in co-culture with hRPTEC under low FSS. Scale bars = 25 µm.

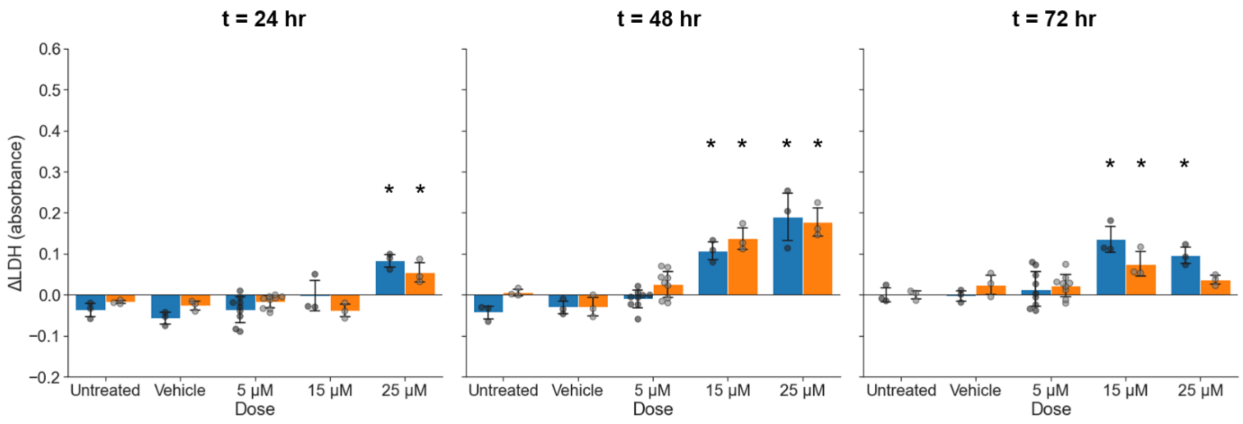

Supplementary Figure S4. LDH release from hMVEC during exposure to cisplatin in co-culture. hMVEC also demonstrated a dose-dependent increase in cell death in response to cisplatin. Media was collected from the top channel (containing hMVEC) of each co-culture device and analyzed for LDH content as described in the Methods. This dataset did not fit a normal distribution based on the Shapiro-Wilk test. *$p<0.05$ based on Kruskal-Wallis with Dunn's post hoc relative to untreated control. There were no significant differences relative to FSS (for corresponding doses and time points) based on the Mann-Whitney U-test.

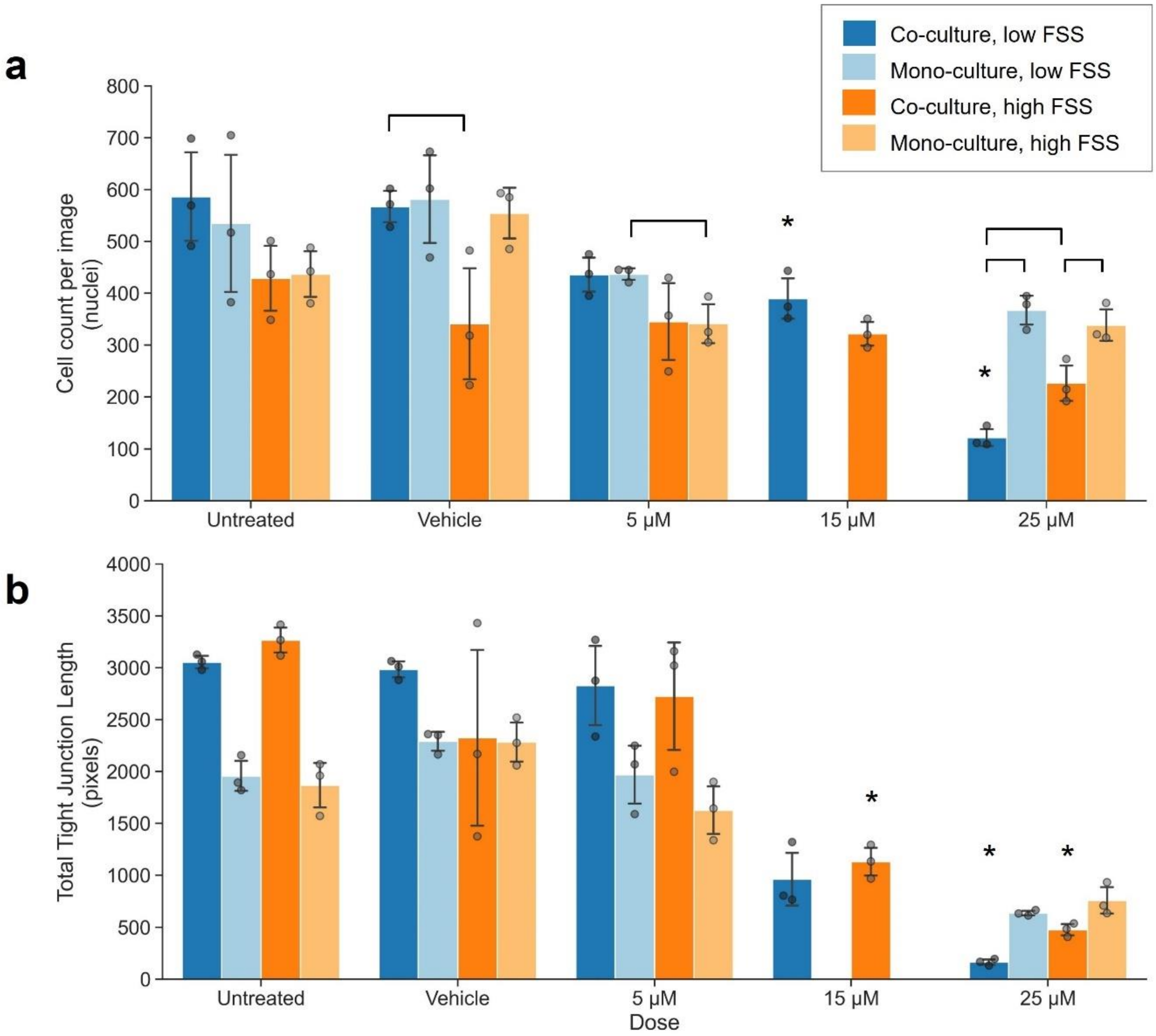

Supplementary Figure S5. Cell counts and total tight junctions after cisplatin exposure. (**a**) Total number of cells per image (equivalent area) based on nuclei identification. (**b**) Total length of tight junctions per image. *p≤0.05 Kruskal-Wallis with Dunn's post-hoc test relative to corresponding untreated culture control. Brackets indicate significant (p≤0.05) comparisons based on two-sided Mann-Whitney U-test.

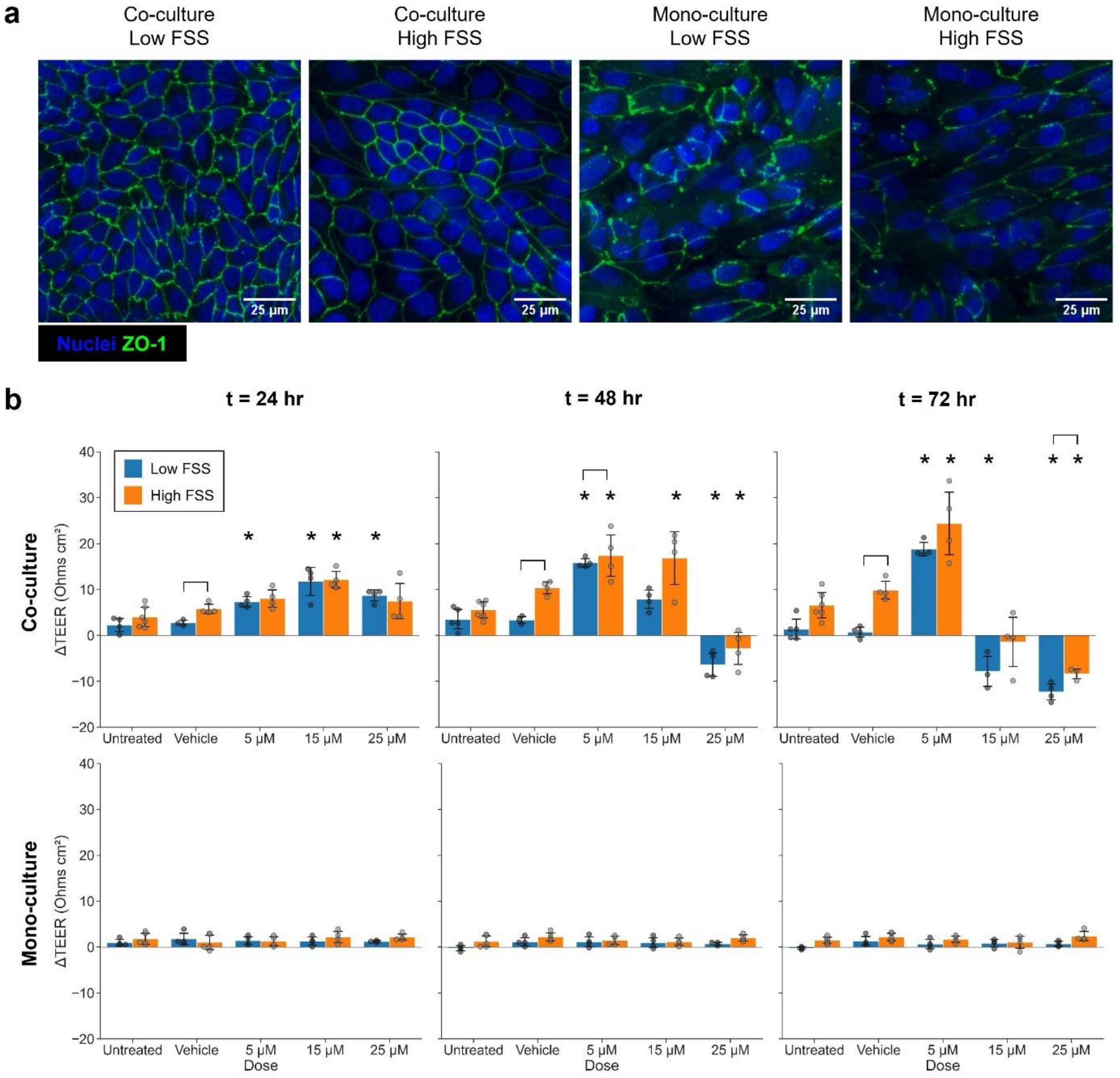

Supplementary Figure S6. Cisplatin response of a different hRPTEC primary donor ('donor #2') measured with TEER. (**a**) Representative images show notable differences in tight junction continuity of hRPTEC donor #2 co-cultured with hMVEC (top row) or in mono-culture (bottom row). Images depict hRPTEC tight junctions (ZO-1, green) and nuclei (Hoechst, blue) after nine days in untreated conditions. (**b**) Net change in TEER relative to baseline (t=0hr) at various time points during cisplatin exposure for hRPTEC-hMVEC co-culture (top row) and hRPTEC mono-culture (bottom row). *$p<0.05$ single-factor ANOVA with Tukey's post hoc relative to untreated control, and brackets indicate significant comparisons ($p<0.05$) based on Students' t-test. n=3-6 devices per condition, N=1 experimental replicate. The co-culture dataset was adequately described by a normal distribution according to the Shapiro-Wilk test of normality ($p=0.074$). No significant differences in TEER were observed for mono-cultures. hRPTEC donor #2 were seeded in the bottom channel, and for co-culture, hMVEC were seeded in the top channel. 'Low FSS' indicates 0.01 dyn/cm^2 in the bottom channel and 0.70 dyn/cm^2 in the top channel. 'High FSS' indicates 0.70 dyn/cm^2 in both channels.

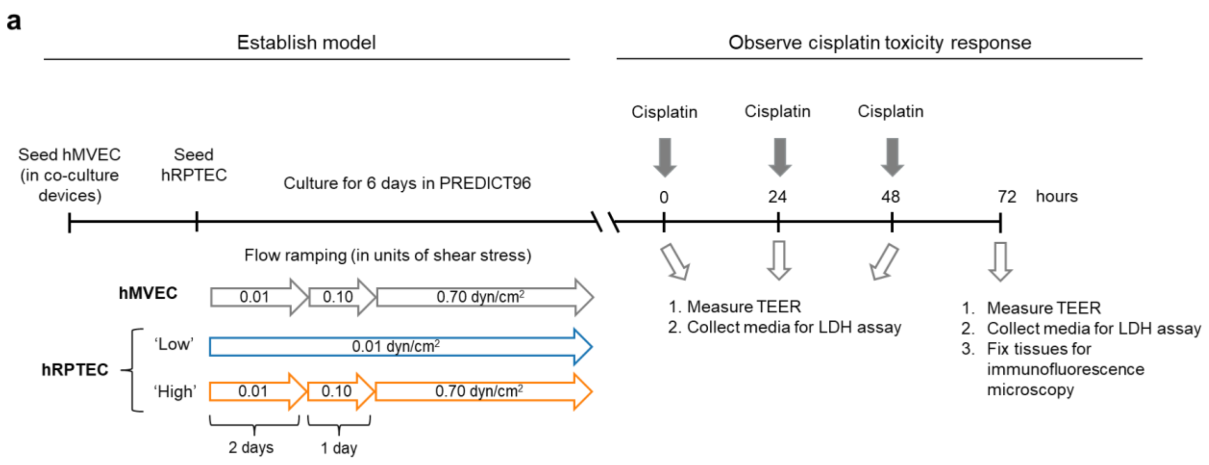

Supplementary Figure S7. Experimental timeline. Timeline of tissue establishment and cisplatin toxicity testing showing cell seeding events, flow ramping, cisplatin dosing events, and time points for TEER and LDH measurements.

a

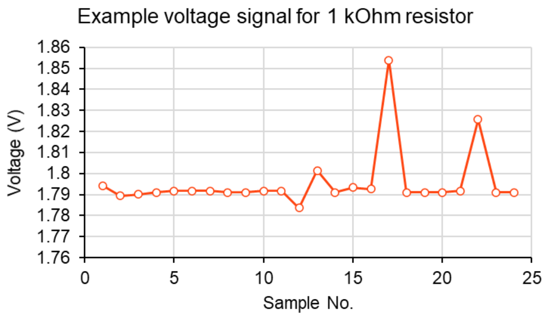

	V	Ohms	Ohms·cm^2
**median**	1.792	38.18	1.413
**mean**	1.795	39.19	1.450
**stdv**	0.014	3.9	0.14

b

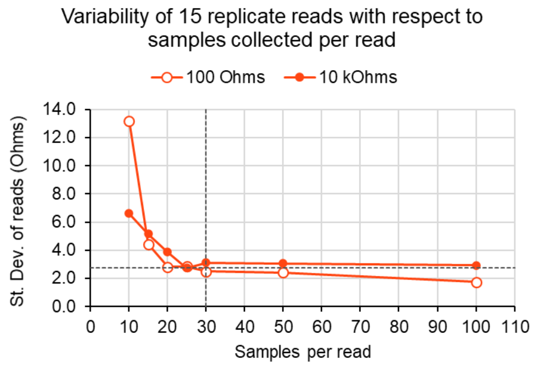

	Ohms	Ohms·cm^2
**Variability at 30 samples per read (standard deviation)**	2.75	0.10

c

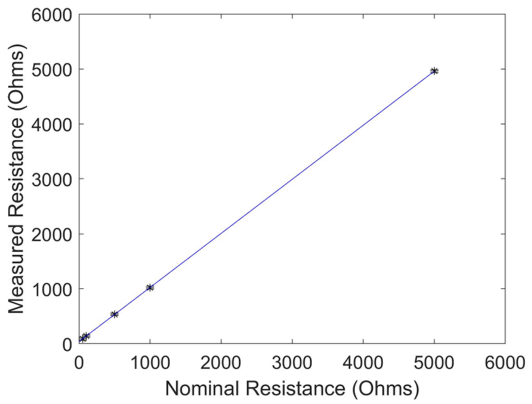

Supplementary Figure S8. Rapid TEER system characterization. (**a**) The voltage signal for known resistors was characterized by random noise, so we used the median value for TEER calculations since it better represented the true signal. (**b**) Measurement precision of the rapid TEER system was evaluated by comparing the standard deviation of 15 replicate reads for known resistors at the high and low ends of the measurable range. With 30 samples per read, the approximate precision of the system was 0.10 Ohms cm^2. (**c**) We evaluate measurement accuracy using known resistors across a range relevant for the PREDICT96 kidney model and found good agreement between nominal and measured resistance.

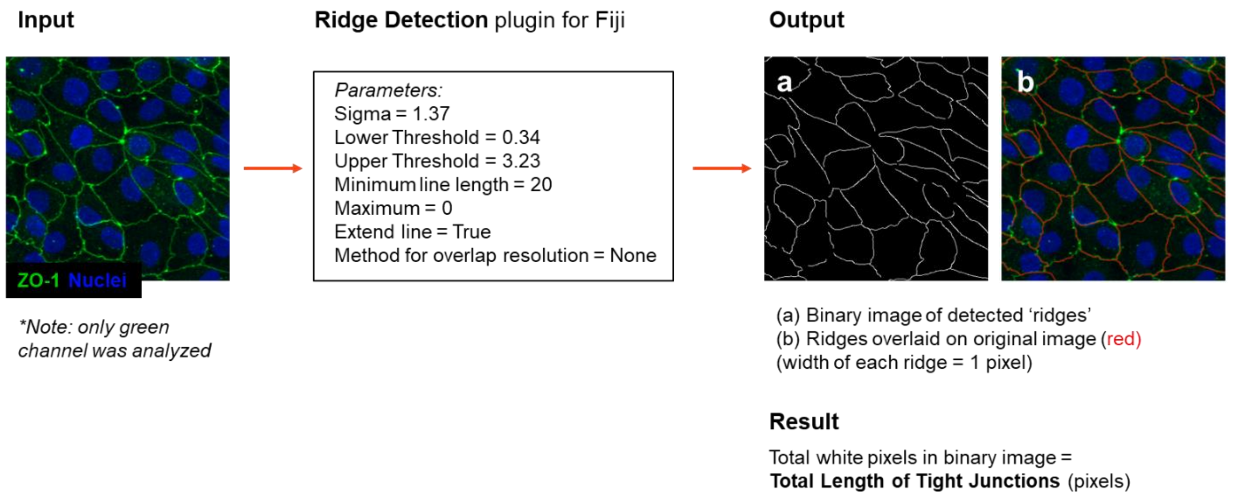

Supplementary Figure S9. Tight junction image analysis workflow. Tight junction expression was analyzed using the Ridge Detection plugin in Fiji with the indicated parameters and confocal immunofluorescence images of tissue stained for ZO-1 (green). The plugin returned a binary image of detected ridges (reduced to a 1-pixel width) and white pixels were counted to determine the total length of tight junctions in the image.

a

Shapiro-Wilk Test	W	*p* value	Follows normal
Change in LDH Release	0.9564	0.0000	No
Change in TEER	0.9558	0.0000	No
Normalized tight junction length	0.9657	0.1240	Yes
Cell count per TJ image	0.9851	0.7382	Yes
Total tight junction length	0.9444	0.0143	No

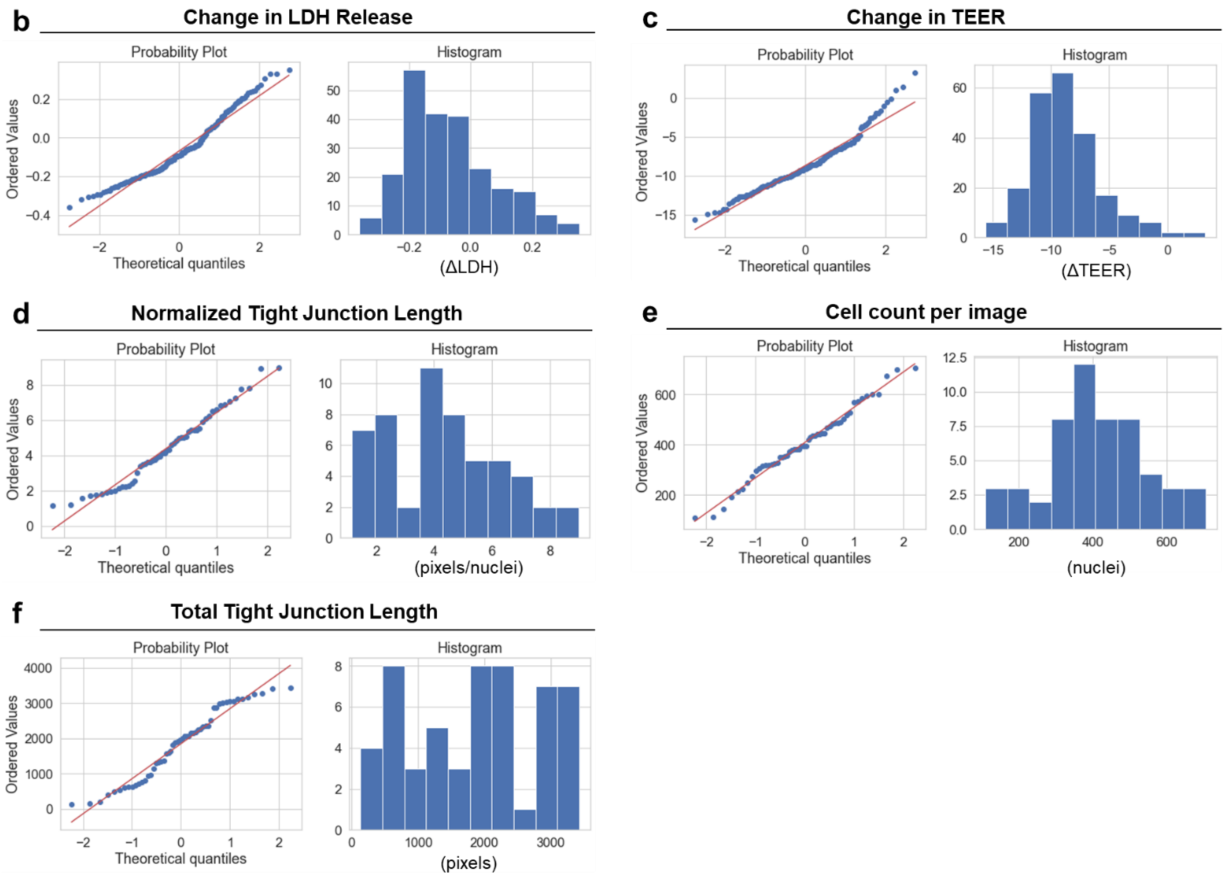

Supplementary Figure S10. Dataset normality assessment. We evaluated whether the experimental datasets were well-modeled by a normal distribution for the purposes of statistical testing. (**a**) Test statistics (W) and *p* values for the Shapiro-Wilk Normality Test by dataset. (**b**-**e**) Probability plots and histograms of datasets. Based on Shapiro-Wilk test and graphical inspection, we assumed that normalized tight junction length (d) cell count (e) were well-modeled by a normal distribution so parametric tests (single-factor ANOVA and Students' t-test) were used to evaluate statistical significance. Non-parametric statistical tests (Kruskal-Wallis and Mann-Whitney U-test) were applied for the remaining datasets (b,c,f).
